# Vacuum-Assisted Interfacial Polymerization Technique for Enhanced Pervaporation Separation Performance of Thin-Film Composite Membranes

**DOI:** 10.3390/membranes12050508

**Published:** 2022-05-10

**Authors:** Marwin R. Gallardo, Micah Belle Marie Yap Ang, Jeremiah C. Millare, Shu-Hsien Huang, Hui-An Tsai, Kueir-Rarn Lee

**Affiliations:** 1 R&D Center for Membrane Technology, Department of Chemical Engineering, Chung Yuan Christian University, Taoyuan 32023, Taiwan; marwin.gallardo95@gmail.com (M.R.G.); huian@cycu.edu.tw (H.-A.T.); 2 School of Chemical, Biological and Materials Engineering and Sciences, Mapúa University, Manila 1002, Philippines; jcmillare@mapua.edu.ph; 3 Department of Chemical and Materials Engineering, National Ilan University, Yilan 26047, Taiwan; 4 Research Center for Circular Economy, Chung Yuan Christian University, Taoyuan 32023, Taiwan

**Keywords:** thin-film composite membranes, pervaporation, interfacial polymerization, polyamide

## Abstract

In this work, thin-film composite polyamide membranes were fabricated using triethylenetetramine (TETA) and trimesoyl chloride (TMC) following the vacuum-assisted interfacial polymerization (VAIP) method for the pervaporation (PV) dehydration of aqueous isopropanol (IPA) solution. The physical and chemical properties as well as separation performance of the TFC_VAIP_ membranes were compared with the membrane prepared using the traditional interfacial polymerization (TIP) technique (TFC_TIP_). Characterization results showed that the TFC_VAIP_ membrane had a higher crosslinking degree, higher surface roughness, and denser structure than the TFC_TIP_ membrane. As a result, the TFC_VAIP_ membrane exhibited a higher separation performance in 70 wt.% aqueous IPA solution at 25 °C with permeation flux of 1504 ± 169 g∙m^−2^∙h^−1^, water concentration in permeate of 99.26 ± 0.53 wt%, and separation factor of 314 (five times higher than TFC_TIP_). Moreover, the optimization of IP parameters, such as variation of TETA and TMC concentrations as well as polymerization time for the TFC_VAIP_ membrane, was carried out. The optimum condition in fabricating the TFC_VAIP_ membrane was 0.05 wt.% TETA, 0.1 wt% TMC, and 60 s polymerization time.

## 1. Introduction

With the increasing demand for energy, the depletion of natural fossil fuel resources, and increasing greenhouse gas emissions, scientists have been exploring the possibility of utilizing alternative energy sources [1,2]. IPA, a potential biofuel, can be used as a gasoline substitute [3]. Common ways to produce IPA are either by the hydration of propene or biomass fermentation [4,5]. To obtain IPA with high purity, removal of water from the byproduct of these processes is a key step, which is energy consuming with the conventional distillation technique. Moreover, IPA and water form an azeotropic mixture at 80.37 °C, making the separation process difficult. Membrane separation technology holds great potential owing to the advantages of overcoming these problems [6,7].

PV is a novel and efficient membrane-based separation process that can be used for alcohol dehydration because of its low cost, ease of operation and it occupies less space [8,9,10]. The separation process is governed by solution-diffusion theory for mass transfer through the membranes. Hence, a selective layer with high hydrophilicity is beneficial for water permeation. Various kinds of hydrophilic polymers have been used in PV membrane fabrication to achieve high water flux. Examples include chitosan [11,12,13], polyvinyl alcohol [14,15,16], polyelectrolyte complex [17,18], and sodium alginate [19,20]. Nevertheless, these are susceptible to plasticization with water, which deteriorates performance. Crosslinking is an established method to reduce the swelling of membranes. However, this would result to trade-off phenomenon between flux and selectivity. Thus, an effective strategy to overcome this problem is to prepare a composite membrane with a thin and dense selective layer, also known as TFC membranes.

TFC membranes have attracted considerable attention, as previous works have already been demonstrated its high separation performance in the dehydration of alcohols [21,22]. A TFC membrane is prepared by synthesizing a thin active layer on the surface of porous support through IP—a polycondensation reaction between monomers dissolved in immiscible phases [23]. Usually, the diamine monomer is dissolved in water, whereas the acyl chloride monomer is dissolved in *n*-hexane. The key steps to prepare polyamide though conventional IP are as follows: (1) immersion of membrane support in aqueous diamine solution; (2) removal of the excess aqueous solution on the surface of membrane support; then, (3) pouring of hexane solution containing acyl chloride on the surface of membrane support to form a polyamide layer. Several IP parameters can be tuned to regulate the surface properties of the polyamide separation layer. These includes the type of membrane support, kind and concentration of monomers, variation of solvents, incorporation of additives, and adopting different IP method [24,25,26,27,28].

Vacuum-suction filtration, a simple and established method of membrane preparation, has been used to deposit nanomaterials dispersions into the porous substrate surface [29,30]. From the perspective of fabricating TFC or thin film nanocomposite (TFN) membranes, it can be utilized not only to effectively deposit the 2D nanomaterial on the membrane support, but also to ensure the homogenous distribution of amine monomers on the surface of the membrane support before initiating the IP reaction [31,32]. Previous works have shown compelling evidence of improving the morphology of the polyamide active layer with this kind of method [32,33,34]. However, as most of the studies conducted are applied to nanofiltration separation, the effects of this technique in PV separation have rarely been demonstrated.

Therefore, the aim of this work was to explore the use of (VAIP) technique for fabricating a TFC membrane for the PV dehydration of aqueous isopropanol solution. In this study, the physicochemical properties and performance of TFC membranes prepared using the VAIP technique were compared with the widely used TIP method. Moreover, the effect of different IP parameters, such as TETA concentration, TMC concentration, and polymerization time on the separation performance of membranes was also investigated.

## 2. Materials and Methods

### 2.1. Materials

Polyacrylonitrile (PAN) polymer was supplied by Tong-Hwa Synthetic Fiber Co. Ltd., Taipei, Taiwan. *n*-methyl-2-pyrrolidone (NMP), the solvent used to dissolve PAN, was obtained from Tedia Company Inc., Fairfield, OH, USA. NaOH, which was used for hydrolysis of PAN, was delivered by Showa Chemical Co., LTD., Tokyo, Japan. Monomers used to form the IP layer, TETA and TMC, were purchased from Merck Co., Darmstadt, Germany, and Tokyo Chemical Industry Co., Ltd., Tokyo, Japan, respectively. Distilled water, which was used as an aqueous phase solvent, was obtained from the laboratory. Reagent-grade *n*-hexane, which was purchased from Tedia Company Inc., Fairfield, OH, USA, was used as an organic phase solvent. Fluorescein sodium salt was acquired from Sigma-Aldrich Co., Burlington, MA, USA. IPA, which was used as feed solution for PV test, was purchased from Echo Chemical Co. Ltd., Miaoli, Taiwan. Liquid nitrogen and helium were purchased from Ming Yang Special Gas Co., Ltd., Taoyuan, Taiwan.

### 2.2. Preparation of Modified PAN (mPAN) Porous Membrane Supports

Porous modified PAN support was prepared similar to our previous work [35]. A polymeric solution of 15 wt% PAN in NMP was casted into a glass plate covered with nonwoven polyester using a casting knife with a gap of 200 μm. Next, the plate was immediately immersed in a water bath at room temperature to induce the coagulation and precipitation of PAN from the solution. The resulting flat and porous PAN membrane was left in a water bath for 24 h to remove the residual NMP and then stored in fresh water.

The PAN support was then hydrolyzed to increase its hydrophilicity, thereby enhancing the absorption of aqueous amine solution on its surface. In detail, PAN membranes were first immersed for 30 min into 2 M NaOH solution preheated at 50 °C. Membranes were then washed with DI water thoroughly until the pH of rinsed water turns neutral (pH = 7). The resulting hydrolyzed PAN (denoted as mPAN) membranes were stored in DI water for future use. The pore size of the PAN and mPAN support are presented in Table S1, where both supports show a pore size of approximately 30 nm.

### 2.3. Fabrication of TFC Membranes

**Figure 1:**
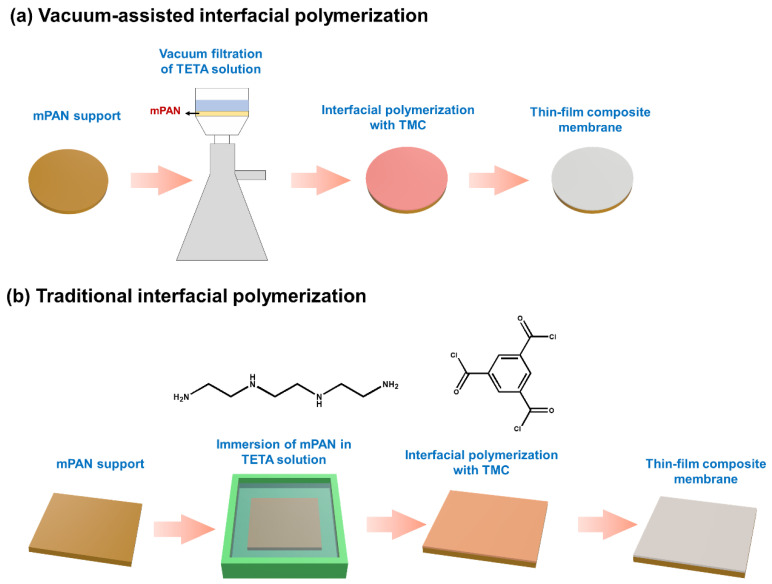
Schematic diagram for preparing (**a**) TFC_VAIP_ and (**b**) TFC_TIP_ membrane.

TFC membranes were prepared by VAIP (Figure 1a). In brief, an mPAN membrane with an effective area of 8.55 cm^2^ was clamped in the vacuum filtration device, followed by flushing of 10 mL DI water through vacuum filtration at a reduced pressure 0.8 bar. Subsequently, 10 mL of 0.05 wt% aqueous TETA solution was poured onto the membrane surface, then vacuum filtrated until the entire amount of solution was removed. Then, 10 mL of 0.5 wt% TMC in *n*-hexane solution was contacted with the amine-saturated mPAN membrane for 1 min, followed by pouring away the organic solution from the membrane. The resulting polyamide membrane was washed with methanol to remove the unreacted monomers and then air dried overnight.

TFC PV membrane was also fabricated using the TIP method for comparison (Figure 1b). In the laboratory-made IP module, the mPAN membrane was fixed. Then, 0.05 wt% of aqueous TETA phase solution was poured into the surface of the membrane to saturate it for 1 min, after which the excess solution was removed by air gun. Next, an organic phase solution containing 0.5 wt% TMC/*n*-hexane solution was poured into the surface of the saturated membrane for 1 min contact time. The as-prepared TFC membranes were washed with methanol and air-dried overnight to use for further tests. The prepared TFC membranes were designated as TFC_TIP_ and TFC_VAIP_ where the subscript corresponds to the IP method (TIP or VAIP) used.

### 2.4. Evaluation of Diamine Monomer Distribution in Porous Support

The laser confocal scanning microscopy (LCSM) technique was adopted [36] to observe the diamine monomer distribution on mPAN surface. An aqueous solution containing 0.05 wt% TETA and 0.005 wt% fluorescein sodium salt was poured into the vacuum filtration device and filtered through the mPAN support under a reduced pressure of 0.8 bar. The mPAN support was scanned on a laser confocal microscope (Nikon A1R, Japan) at an excitation wavelength of 488 nm. For comparison, another sample was prepared under the same condition but using the TIP: the mPAN membrane was immersed in aqueous solution (TETA + fluorescein) for 1 min, then the residual solution on the membrane support surface was removed by using an air gun.

FTIR mapping was also used to observe the absorption of TETA monomers on the mPAN substate. The procedure is similar to the preparation of confocal microscopy samples described above, but without the addition of fluorescein sodium in the aqueous solution. The dried mPAN saturated with TETA was put into the sample stage of the FTIR spectroscope coupled with a microscope (Jasco FTIR-6700 and IRT-5200, Japan).

### 2.5. Membrane Characterization

The surface chemical composition of membranes was measured using attenuated total reflectance-Fourier transform infrared (ATR-FTIR) spectroscopy (Perkin Elmer Spectrum 100 FTIR Spectrometer, Waltham, MA, USA) and X-ray photoelectron spectroscopy (XPS, VG K-alpha ThermoFisher Scientific, Inc., Waltham, MA, USA). Membrane morphology and surface roughness were observed using field emission scanning electron microscopy (FESEM, S-4800, Hitachi Co., Tokyo, Japan) and atomic force microscopy (AFM, NanoScope^®^ V, Bruker, Billerica, MA, USA), respectively. The surface wetting characteristics of the membranes were measured using an automatic interfacial tensiometer (PD-VP Model, Kyowa Interface Science Co., Ltd., Niiza City, Saitama, Japan).

### 2.6. Membrane Performance Test

The PV separation of 70 wt% aqueous isopropanol solution at an operating temperature of 25 °C was measured using a laboratory-scale setup [37]. Steady-state conditions were first established for 30 min prior to sampling. The permeate was collected in cold traps immersed in liquid nitrogen. The flux of permeate was calculated using Equation (1):

(1) $$J=\frac{m}{A\times t}\;\;$$

where *J* is the permeation flux in g∙m^−2^∙h^−1^, m is the permeate sample weight in grams, *A* is the effective area of the membrane (4.91 × 10^−4^ m^2^), and *t* is the sampling time in hours. The composition of permeate was determined using a gas chromatography analyzer (China Chromatography Personal GC 1000, China Chromatography Co., Ltd., Taipei, Taiwan).

Separation factor (*α*) and PV separation index (PSI) were determined using Equations (2) and (3), respectively:

(2) $$\alpha =\frac{Y_{W}/Y_{A}}{X_{W}/X_{A}}\;\;\;\;\;\;\;\;\;\;\;\;\;\;\;\;\;$$

(3) $$PSI=J\times \left(\alpha -1\right)\;\;\;\;\;\;\;$$

where *X_W_* and *X_A_* are the respective concentrations of water and isopropanol on the feed side, and *Y_W_* and *Y_A_* are the respective concentrations of water and isopropanol on the permeate side.

## 3. Results and Discussion

### 3.1. Chemical Structure and Morphology of TFC Membranes

**Figure 2:**
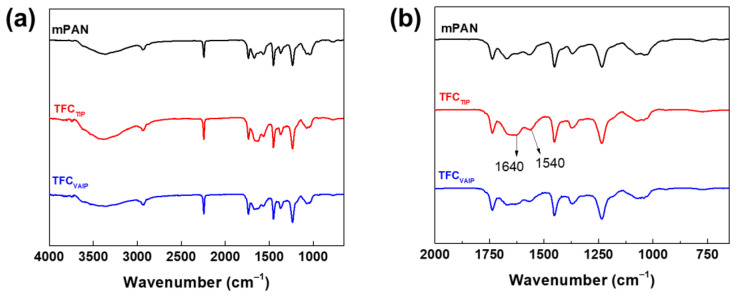
(**a**) FTIR spectra of mPAN, TFC_TIP_, and TFC_VAIP_ membranes (**b**) enlarged view of the spectra (*x*-axis: 2000 to 650 cm^−1^).

Figure 2 presents the ATR-FTIR spectra of mPAN and TFC membranes. Compared with the mPAN reference spectrum, the spectra of TFC membranes from the reaction of TETA and TMC generated new peaks at 1640 cm^−1^, corresponding to amide I (C=O) [25,38,39]. However, it was difficult to observe the peak at 1540 cm^−1^ corresponding to the amide II (N–H) of both TFC_TIP_ and TFC_VAIP_ membranes, as the polyamide layer was probably very thin, and it overlapped with the spectra of mPAN.

**Figure 3:**
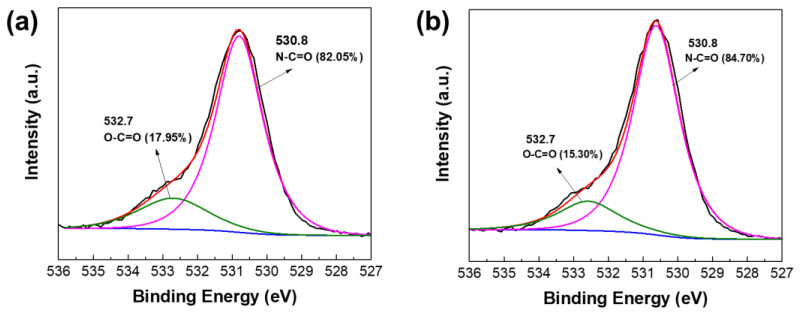
XPS spectra for O1s of (**a**) TFC_TIP_, and (**b**) TFC_VAIP_ membranes.

XPS analysis was carried out to further analyze the surface chemistry of TFC membranes. Figure 3 shows the O1s spectra of the membranes. There are two distinct peaks that can be observed, namely O–C=O and N–C=O at binding energies of 532.7 and 530.8 eV, respectively. The oxygen in the N–C=O bond was from the amide group formed from the IP reaction of TETA with TMC, whereas those in the O–C=O bond originated from the carboxyl groups from the hydrolysis of the remaining acyl chloride groups of TMC on the formed polyamide [40]. It is known that higher N–C=O/O–C=O means a higher crosslinking degree of the membrane [41]. The result showed that TFC_VAIP_ had higher N–C=O/O–C=O ratio (5.53) than TFC_TIP_ membrane (4.57), meaning it had a denser polyamide layer. The vacuum-assisted method can enrich diamine monomers on the mPAN support, which ensures that there are enough diamine monomers to react with TMC, thereby enhancing the cross-linking degree of the resulting polyamide layer [42,43]. A denser structure would be beneficial to the high separation efficiency of the membrane. This result also confirms the presence of the polyamide layer on the surface of both membranes.

**Figure 4:**
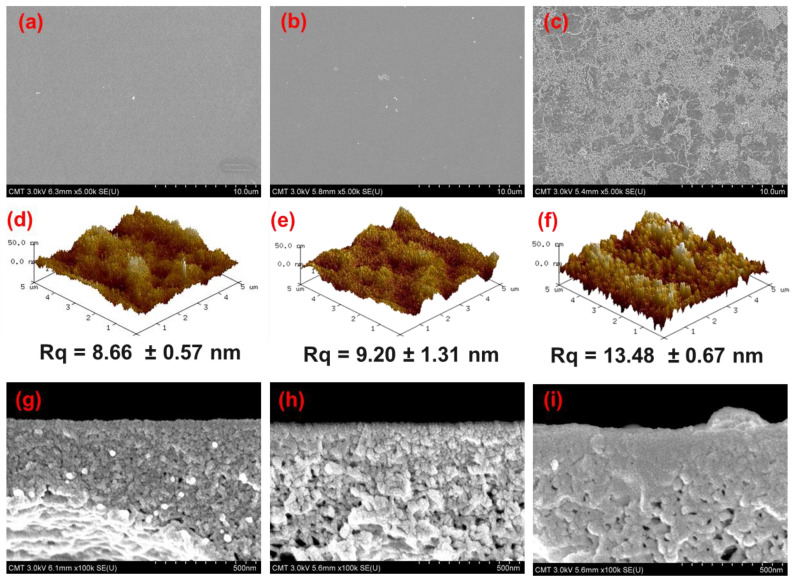
SEM and AFM surface images of (**a**,**d**,**g**) mPAN, (**b**,**e**,**h**) TFC_TIP_, (**c**,**f**,**i**) TFC_VAIP_ membranes.

Figure 4 presents the morphology and roughness of mPAN and TFC membranes. mPAN support showed a smooth and porous structure (Figure 4a). After depositing the polyamide layer through the TIP method, the TFC_TIP_ membranes had a smooth surface with small bumps distributed on it (Figure 4b). In contrast, a wrinkled structure can be observed on surface TFC_VAIP_ membrane (Figure 4c), exhibiting a rougher surface than the TFC_TIP_. It can be speculated that the rougher surface might be due to the faster polymerization rate during the membrane fabrication through VAIP. In this method, mPAN support was able to absorb more amine monomers than the TIP method.

The 3D AFM images of membranes are presented (Figure 4g–i). The surface roughness exhibited a decreasing order as follows: TFC_VAIP_ (Rq = 13.48 ± 0.67 nm) > TFC_TIP_ (Rq = 9.20 ± 1.31 nm) > mPAN (Rq = 8.66 ± 0.57 nm). These results were consistent with the FESEM morphology of the membranes. Furthermore, the cross-sectional images of mPAN and TFC membranes are provided to depict the structure of the polyamide layer. It can be observed that the thickness of the selective layer for both the TFC_TIP_ and TFC_VAIP_ was hard to distinguish (Figure 4h,i), implying that the polyamide layers were very thin. In addition, it can be noticed that the morphology of TFC_VAIP_ had fewer pores than the TFC_TIP_, suggesting the intrusion of polyamide into the pores of mPAN.

**Figure 5:**
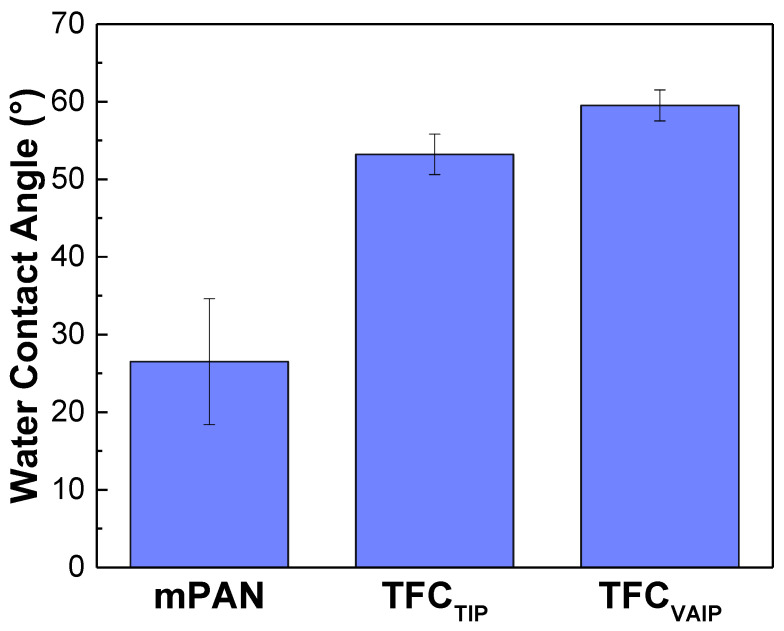
Water contact angle of membranes after 1 min.

Figure 5 indicates the water contact angle of mPAN, TFC_TIP_, and TFC_VAIP_ membranes. The water contact angle showed an increasing order as follows: mPAN (26.5 ± 8.1°) < TFC_TIP_ (53.2 ± 2°) < TFC_VAIP_ (59.5 ± 2°). A lower water contact angle implies that the membrane has a hydrophilic surface. The establishment of a polyamide layer on mPAN support increased the water contact angle for both the TFC_TIP_ and TFC_VAIP_ membranes. Based on the chemical structure, TFC membranes have a benzene group on the surface, rendering the membrane more hydrophobic. Meanwhile, the TFC_VAIP_ membrane had a water contact angle of 59.5 ± 2.0°, which was relatively higher than the TFC_TIP_, even though it had a higher surface roughness. This was because of the presence of free hydrophilic carboxyl groups on the surface of TFC_TIP_, as revealed in FTIR (Figure 2) and XPS data (Figure 3), dominating the overall hydrophilicity.

### 3.2. Diamine Monomer Spreading on mPAN Support

**Figure 6:**
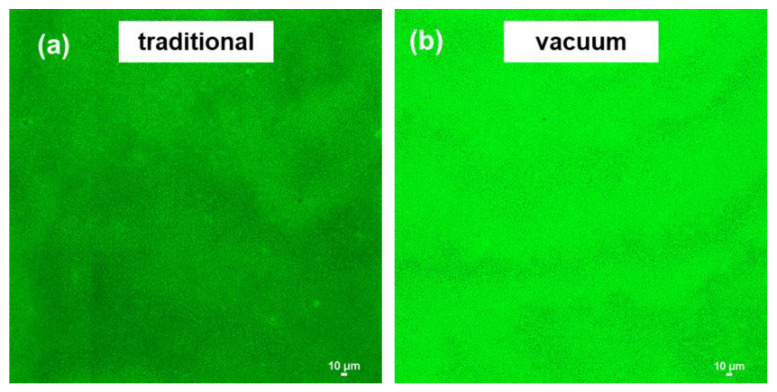
2D LSCM of mPAN microporous support saturated with TETA aqueous monomer though (**a**) traditional (immersion) and (**b**) vacuum filtration method.

It has been reported that employing a different method of saturating the porous membrane support before IP would affect the distribution of diamine monomer, thereby influencing the formation of the polyamide layer and its morphology [34]. Figure 6 presents the distribution of the TETA monomer on mPAN from 2D LCSM. The distribution behavior of TETA in mPAN support was described through the intensity of fluorescence. The porous support saturated using the vacuum filtration method emits stronger and more uniform green fluorescence than the one prepared using the traditional method, indicating that more diamine monomers are absorbed by mPAN.

**Figure 7:**
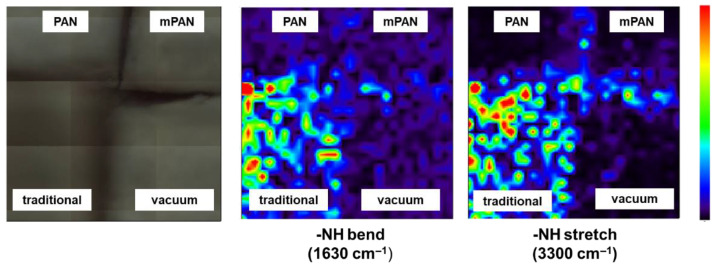
Microscopic images of membranes and their corresponding FTIR maps at specific wavenumbers.

To corroborate the above results, FTIR mapping was carried out. The analysis was performed at 1630 cm^−1^ and 3300 cm^−1^, corresponding to the -NH bending and stretching of the TETA monomer, respectively. The color code used was related to the intensity of this band: from dark blue (low intensity, indicating an absence of amine group of TETA) to red (high intensity, indicating presence of amine group of TETA). Figure 7 implies that mPAN saturated using vacuum filtration (lower right quadrant) have a deep blue color. In contrast, the mPAN saturated with the traditional/immersion method (lower left quadrant) have predominantly green color with reddish-orange spots. This indicates that TETA monomers were mainly present in the surface of mPAN saturated through the traditional method, whereas TETA was difficult to detect on the vacuum filtration method.

The results from 2D LSCM and FTIR mapping were contradictory. We deduced that, in the case of the traditional method, the amount of TETA monomers on the surface of the mPAN was enough to be detected by FTIR mapping. Meanwhile, for the vacuum method, more TETA monomers were enriched inside the mPAN support that cannot be detected by FTIR mapping but can be easily detected by the 2D LSCM. It is worthy to mention that confocal microscopy has a high penetration depth on samples, which is around 100 µm [44].

**Figure 8:**
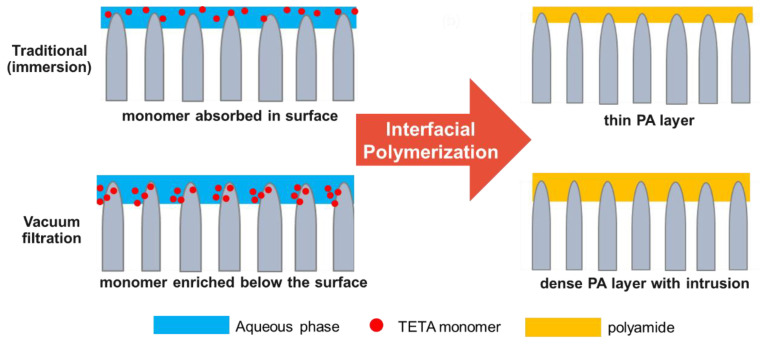
Scheme of distribution of TETA monomers on mPAN support and formation of PA layer through traditional IP and vacuum-assisted IP.

Based on the above analysis, the schematic of diamine spreading and the formation of polyamide layer was proposed and is presented in Figure 8. Compared with the conventional technique, the VAIP method would enrich the TETA monomers mostly under the mPAN surface, which was near the reaction boundary. As a result, there would be an optimum amount of TETA to react with TMC, leading to a denser structure, as shown in XPS data (Figure 3).

### 3.3. Pervaporation Performance of TFC Membranes

**Figure 9:**
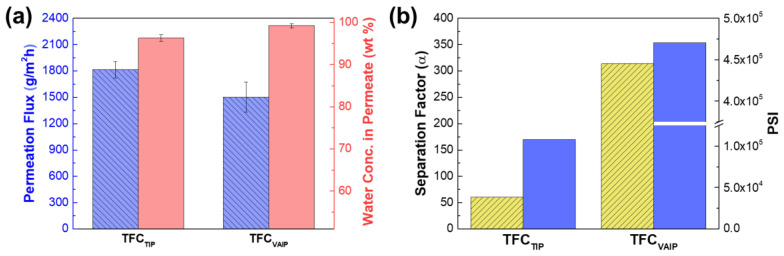
(**a**) Pervaporation performance of TFC_TIP_ and TFC_VAIP_ membranes and (**b**) separation factor and PSI. Feed =70 wt% isopropanol; feed temperature = 25 °C.

Figure 9 summarizes the PV performance of membrane prepared using TIP and VAIP method in dehydrating 70 wt% aqueous isopropanol solution at 25 °C. The TFC_TIP_ membrane had a higher permeation flux of 1815 ± 93 g∙m^−2^∙h^−1^ and lower water concentration in permeate of 96.28 ± 0.76 wt% (α = 61), than that of the TFC_VAIP_ membrane (permeation flux of 1504 ± 169 g∙m^−2^∙h^−1^ water concentration in permeate of 99.26 ± 0.53 wt% (α = 314)). Because in the VAIP method, more amine was adsorbed on the mPAN support (Figure 6), a dense polyamide layer would form, resulting in higher separation efficiency.

According to the solution-diffusion theory, the enhancement of separation can be ascribed to the solution (or sorption) process and diffusion process [45,46,47]. However, the surface hydrophilicity of the TFC_VAIP_ membrane was relatively lower than that of the TFC_TIP_ as presented in the water contact angle data (Figure 5). Hence, it was certain that the diffusion process was the main reason for the obvious enhancement of the separation and not the sorption process. The dense polyamide layer greatly contributed to the improvement of the diffusion selectivity of the TFC_VAIP_ membrane, which provides mass transfer resistance against larger sized IPA molecules while allowing the passage of smaller sized water molecules. It was noteworthy to mention that the decrease in hydrophilicity of the TFC_VAIP_ membrane might contributed to the decline in permeation flux and its sorption selectivity, but it was the diffusion selectivity that dominated. TFC_VAIP_ membrane had a higher separation factor that was almost five times that of the TFC_TIP_ membrane. This result demonstrated the potential of VAIP in fabricating TFC membranes with high separation performance.

### 3.4. Optimization of IP Conditions for TFC_VAIP_ Membranes

**Figure 10:**
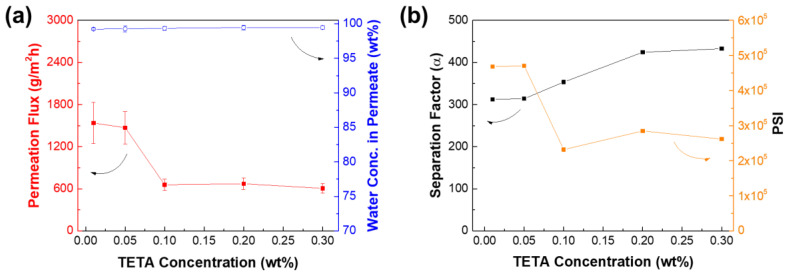
Effect of diamine monomer concentration on TFC_VAIP_ membrane (**a**) pervaporation performance and (**b**) separation factor and PSI. Feed =70 wt% isopropanol; feed temperature = 25 °C. IP conditions: 0.5 wt% TMC, 60 s reaction time.

Getting the optimum concentration of monomers is one of the key steps in fabricating high performance TFC membranes. Figure 10 illustrates the membrane performance as a function of the TETA monomer concentration from 0.01 to 0.3 wt%. The increase in diamine monomer concentration would result in a decrease in permeation flux, while the water concentration in permeate increases. The optimum concentration of TETA was determined at 0.05 wt%, wherein the permeation flux was 1471 ± 230 g∙m^−2^∙h^−1^ and water concentration in permeate of 99.26 (α = 314). Beyond that, the membrane performance levelled off. This is because the polyamide layer was already dense enough that the amine monomers could not penetrate to react with TMC in the organic phase solution. Consequently, this resulted in limiting the growth of the polyamide active layer.

**Figure 11:**
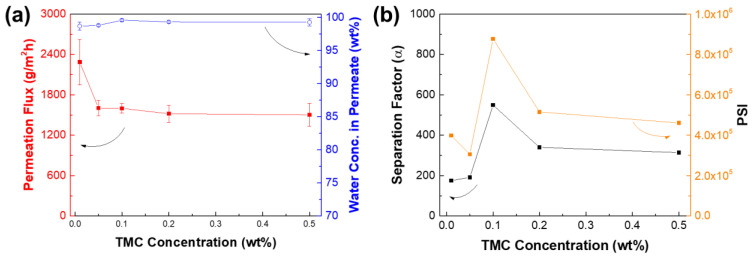
Effect of triacyl chloride monomer concentration on TFC_VAIP_ membrane (**a**) performance and (**b**) separation factor and PSI. Feed = 70 wt% isopropanol; feed temperature = 25 °C. IP conditions: 0.05 wt% TETA.

Figure 11 reveals the performance of membranes as a function of different TMC concentration from 0.01 to 0.5 wt%. The increase in TMC concentration also resulted in a decrease in permeation flux, whereas the water concentration in permeate increased, then remained constant. The membrane performance levelled off in terms of the permeation flux and water concentration in permeate at a TMC concentration higher than 0.1 wt%. Hence, the optimum TMC concentration was determined at 0.1 wt%, in which the permeation flux was 1600 ± 73 g∙m^−2^∙h^−1^ and water concentration in permeates of 99.58 (α = 549).

**Figure 12:**
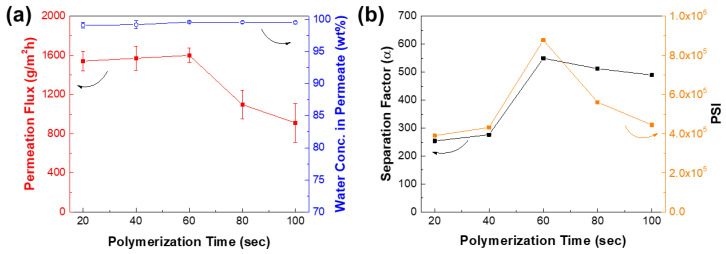
Effect of polymerization time on TFC_VAIP_ membrane (**a**) performance and (**b**) separation factor and PSI. Feed = 70 wt% isopropanol; feed temperature = 25 °C. IP conditions: 0.05 wt% TETA, 0.1 wt% TMC.

The polymerization time also affects the performance of TFC membranes. Generally, the longer the reaction time of monomers, the denser a polyamide layer can be formed. Figure 12 plots the performance of membrane as a function of polymerization time from 20 to 100 s. At the reaction time of 20–60 s, the permeation flux did not significantly change while the water concentration in permeate increased. This is because the low concentration of TETA and TMC monomers can slow down the growth of the polyamide layer, even when the polymerization time was increased. Beyond the reaction time of 60 s, it can be observed that the permeation flux dramatically decreased while the water concentration in permeate remained similar. The separation factor was observed to be the highest at 60 s, suggesting that the membrane started to become dense at this point. Hence, the optimum reaction time was at 60 s, wherein the permeation flux was 1600 ± 73 g∙m^−2^∙h^−1^ and water concentration in permeates of 99.58 (α = 549).

### 3.5. Membrane Stability Test and Comparison with Literature

**Figure 13:**
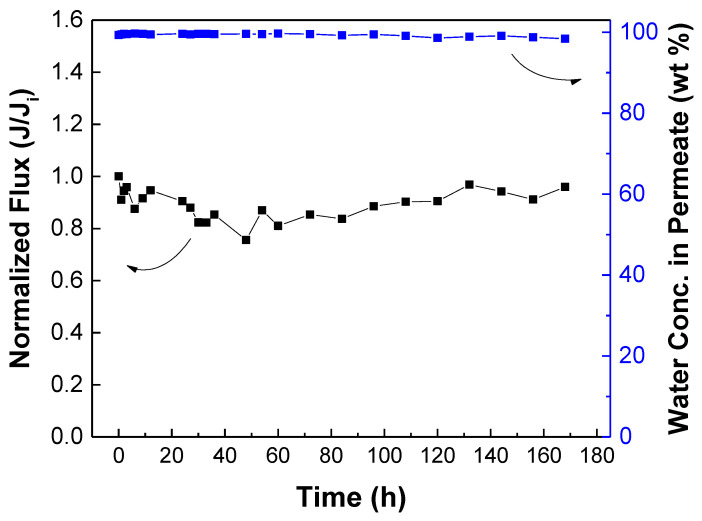
Long-term stability test of TFC_VAIP_ membrane for 168 h. Feed = 70 wt% isopropanol; feed temperature = 25 °C.

The stability of the membrane is important for practical applications. The stability of the TFC_VAIP_ membrane in long-term operation was investigated using 70 wt% aqueous isopropanol solution at 25 °C (Figure 13). During the 168 h operation, the normalized flux showed fluctuations (but it remained close to the initial value) while the water concentration in permeate was maintained at a high level. Thus, the TFC_VAIP_ had stable performance for long-term use. Furthermore, the isopropanol dehydration performance of the TFC_VAIP_ membrane was compared with PV membranes reported in previous literature (Table 1). Its permeation flux and water concentration in permeate were comparable with that of the others. Therefore, TFC_VAIP_ holds great promise as a PV membrane for dehydrating isopropanol/water solutions.

## 4. Conclusions

In this work, we have investigated the potential of using the VAIP technique in fabricating TFC membranes for PV separation. The employment of this method would lead to the enrichment of TETA monomers into the mPAN support, resulting in the successful formation of a polyamide layer with tailored morphology. Characterization results show that the TFC_VAIP_ membrane had a higher crosslinking degree, higher surface roughness, and denser structure than the TFC_TIP_ membrane. Such an improved physicochemical structure polyamide layer renders the TFC_VAIP_ membrane with high efficiency in separating 70 wt% isopropanol/water mixture with permeation flux of 1504 ± 169 g∙m^−2^∙h^−1^, water concentration in permeate of 99.26 ± 0.53 wt%, and separation factor of 314 that is almost five times higher than the membrane prepared through TIP. Moreover, the optimization of IP parameters, such as the variation of TETA and TMC monomer concentrations as well as polymerization time for the TFC_VAIP_ membrane, was carried out. It turns out that the TFC_VAIP_ membrane prepared using 0.05 wt% TETA, 0.1 wt% TMC, and 60 s polymerization time exhibited the optimum performance, with permeation flux of 1600 ± 73 g∙m^−2^∙h^−1^ and water concentration in permeate of 99.58 (α = 549).

## Figures and Tables

**Table 1:** Comparison of pervaporation performance of various membranes for isopropanol/water solution.

Membrane	IPA in Feed (wt%)	Temperature (°C)	Permeation Flux (g∙m^−2^∙h^−1^)	Water Conc. in Permeate (wt%)	Reference
PDAA/PVDF	70	25	95.7	2411	[48]
CR-PBz/PEI	70	30	357	100	[49]
CS-PVA/PVDF HF	90	30	70	98.7	[50]
Chitosan-HMDI/PSf	70	30	1600	97.1	[11]
PA/eGO/PAN	90	30	1670	99.15	[51]
HEC/SA/PAN	70	22	1212	95.54	[52]
PA/PAN HF	90	25	419	96.60	[53]
TFC_VAIP_	70	25	1600	99.58	This work

HEC: hydroxyethyl cellulose; HMDI: hexamethylene diisocyanate; HF: hollow fiber; PBZ: polybenzoxazine; PDAA: polydopamine-amide.

## Supplementary Materials

The following are available online at https://www.mdpi.com/article/10.3390/membranes12050508/s1, Table S1: Pore size of PAN and mPAN membranes.

## List of Symbols

*J*	permeation flux
*J_i_*	initial permeation flux
α	separation factor
*X_W_*	water concentration in feed
*X_A_*	alcohol concentration in feed
*Y_W_*	water concentration in permeate
*Y_A_*	alcohol concentration in permeate
