# Supplementary material for: Vacuum-Assisted Interfacial Polymerization Technique for Enhanced Pervaporation Separation Performance of Thin-Film Composite Membranes
Source: Membranes (Basel). 2022 May 10;12(5):508. doi: 10.3390/membranes12050508 (PMC9144448; doi:10.3390/membranes12050508)
Supplement: Supplementary file 1 [file membranes-12-00508-s001.zip › membranes-1686916-supplementary.pdf]

membranes

Supplementary Materials: Vacuum-Assisted Interfacial Polymerization Technique for Enhanced Pervaporation Separation Performance of Thin-Film Composite Membranes

Marwin R. Gallardo [1], Micah Belle Marie Yap Ang [1,*], Jeremiah C. Millare [2], Shu-Hsien Huang [1,3,*], Hui-An Tsai [1] and Kueir-Rarn Lee [1,4,*]

[1] R&D Center for Membrane Technology, Department of Chemical Engineering, Chung Yuan Christian University, Taoyuan 32023, Taiwan; marwin.gallardo95@gmail.com (M.R.G.); huian@cycu.edu.tw (H.-A.T.)
[2] School of Chemical, Biological, and Materials Engineering and Sciences, Mapúa University, Manila 1002, Philippines; jcmillare@mapua.edu.ph (J.C.M.)
[3] Department of Chemical and Materials Engineering, National Ilan University, Yilan 26047, Taiwan
[4] Research Center for Circular Economy, Chung Yuan Christian University, Taoyuan 32023, Taiwan
* Correspondence: mbmyang@gmail.com (M.B.M.Y.A.); huangsh@niu.edu.tw (S.-H.H.); krlee@cycu.edu.tw (K.-R.L.)

Table S1. Pore size of PAN and mPAN membranes.

Membrane	Pore size (nm)
PAN	29.90 ± 1.56 nm
mPAN	30.21 ± 0.77 nm

The pore size of the PAN and mPAN was measured using ImageJ software from their FESEM images. PAN and mPAN support had approximately 30 nm pore diameter.
